# Structural and functional consequences of buserelin-induced enteric neuropathy in rat

**DOI:** 10.1186/s12876-014-0209-7

**Published:** 2014-12-11

**Authors:** Elin Sand, Bodil Roth, Björn Weström, Peter Bonn, Eva Ekblad, Bodil Ohlsson

**Affiliations:** Department of Clinical Sciences, Division of Internal Medicine Skåne University Hospital, Lund University, Inga Marie Nilssons street 32, S-205 02 Malmö, Sweden; Department of Experimental Medical Science, Neurogastroenterology Unit, BMC B11, Lund University, 221 84 Lund, Sweden; Department of Biology, Functional Biology, Lund University, 221 00 Lund, Sweden; Department of Medicinal Chemistry, CVMD, AstraZeneca, Mölndal, Sweden

**Keywords:** Enteric neuropathy, Enteric subpopulations, Gastrointestinal (GI) tract, Gonadotropin-releasing hormone (GnRH), Luteinizing hormone (LH), Somatostatin

## Abstract

**Background:**

Women treated with gonadotropin-releasing hormone (GnRH) analogs may develop enteric neuropathy and dysmotility. Administration of a GnRH analog to rats leads to similar degenerative neuropathy and ganglioneuritis. The aim of this study on rat was to evaluate the early GnRH-induced enteric neuropathy in terms of distribution of neuronal subpopulations and gastrointestinal (GI) function.

**Methods:**

Forty rats were given the GnRH analog buserelin (20 μg, 1 mg/ml) or saline subcutaneously, once daily for 5 days, followed by 3 weeks of recovery, representing one treatment session. Two weeks after the fourth treatment session, the animals were tested for GI transit time and galactose absorption, and fecal weight and fat content was analyzed. After sacrifice, enteric neuronal subpopulations were analyzed. Blood samples were analyzed for zonulin and antibodies against GnRH and luteinizing hormone, and their receptors.

**Results:**

Buserelin treatment transiently increased the body weight after 5 and 9 weeks (p < 0.001). Increased estradiol in plasma and thickened uterine muscle layers indicate high estrogen activity. The numbers of both submucous and myenteric neurons were reduced by 27%–61% in ileum and colon. The relative numbers of neurons containing calcitonin gene-related peptide (CGRP), cocaine- and amphetamine-related transcript (CART), galanin, gastrin-releasing peptide (GRP), neuropeptide Y (NPY), nitric oxide synthase (NOS), serotonin, substance P (SP), vasoactive intestinal peptide (VIP) or vesicular acetylcholine transporter (VAchT), and their nerve fiber density, were unchanged after buserelin treatment, but the relative number of submucous neurons containing somatostatin tended to be increased (p = 0.062). The feces weight decreased in buserelin-treated rats (p < 0.01), whereas feces fat content increased (p < 0.05), compared to control rats. Total GI transit time, galactose absorption, zonulin levels in plasma, and antibody titers in serum were unaffected by buserelin treatment.

**Conclusions:**

A marked enteric neuronal loss with modest effects on GI function is found after buserelin treatment. Increased feces fat content is suggested an early sign of dysfunction.

**Electronic supplementary material:**

The online version of this article (doi:10.1186/s12876-014-0209-7) contains supplementary material, which is available to authorized users.

## Background

A subgroup of patients treated with gonadotropin-releasing hormone (GnRH) analogs develops enteric neuropathy with reduced relative number of GnRH-containing neurons and dysmotility [1,2], and increased abdominal pain and exacerbation of irritable bowel syndrome (IBS) has been observed in a cohort of GnRH-treated women at follow-up, although no obvious dysmotility was at hand [3]. This knowledge rendered us to set up an experimental rat model to examine the effects on the gastrointestinal (GI) tract of systemic and repeated treatment with the GnRH analog buserelin. About 50% of enteric neurons were lost throughout the GI tract after four treatment periods of buserelin [4,5]. In addition, myenteric ganglia displayed ganglioneuritis [5] and a significant reduction of the relative number of luteinizing hormone (LH) receptor-immunoreactive neurons [4]. In colon, a transient increase in the relative number of vasoactive intestinal peptide (VIP)-immunoreactive myenteric neurons was found after two treatment periods, and increased relative numbers of nitric oxide synthase (NOS)-immunoreactive submucous and myenteric neurons after four treatment periods [4].

The enteric nervous system (ENS) contains a plethora of neurotransmitters, which all participate in the pivotal role of ENS in controlling and modulating GI motility, secretion, and blood flow. The neurotransmitters NOS, pituitary adenylate cyclase-activating peptide (PACAP), purines, somatostatin, and VIP mediate inhibitory transmission, while excitatory transmitters represent acetylcholine, calcitonin gene-related peptide (CGRP), cocaine- and amphetamine-related transcript (CART), galanin, gastrin-releasing peptide (GRP), neurokinin A, neuropeptide Y (NPY), serotonin (5-hydroxytryptamine; 5-HT), and substance P (SP) [6].

There is evidence of a high plasticity of the ENS in response to injurious events in various experimental models, e.g. axotomy, transplantation, hypertrophy, ischemia/reperfusion, and lipopolysaccharide (LPS) challenge [7-11], as well as in diseases [12,13]. Experimental rat models of enteric neuropathy have been described, e.g. neuropathy after cisplatin-, diabetes-, or fat induction [14-16]. Most studies describe pathophysiology and morphological changes in neuropathy, whereas few studies describe any functional consequences on GI motility, nutritional absorption, and intestinal permeability.

The aim of the present study was to describe possible early effects of buserelin-induced enteric neuronal loss on subpopulations of neurons and on body weight, circulating levels of sex hormones and antibodies, GI transit time, feces weight and fat content, nutrient absorption, and epithelial permeability.

## Methods

### Animals

Female Sprague–Dawley rats (n = 40, 170–180 g), purchased from Charles River, Sulzfeld, Germany, were used. The rats were allowed to acclimatize to the climate- and light-controlled animal facility for at least 5 days prior to experimentation. Standard rat chow (4% fat/g) (Lactamin R36, Stockholm, Sweden) and water were supplied at all times. The experimental design was approved by the Animal Ethics Committee, Lund and Malmö, Sweden (M350-12, date of approval: 14.11.12). Animals were used in accordance with the European Communities Council Directive (2010/63/EU) and the Swedish Animal Welfare Act (SFS 1988:534).

### Study design

Twenty-four rats (n = 24) were given 20 μg (1 mg/ml) of the GnRH analog buserelin (Suprefact®, Sanofi-Aventis, Bromma, Sweden) subcutaneously, once daily for 5 days, followed by 3 weeks of recovery, representing one session of treatment (for details see ref no. [4]). The dosage and administration of buserelin are based on previous studies which have shown reliable physiological effects in terms of uterine hypertrophy, without any adverse effects [4,17]. Control animals (n = 16) received saline injections. The animals were weighed prior to inclusion in the study, and weekly in the morning during the study, using an electronic scale. Half of the animals were used to examine vaginal smears. From the other rats (12 buserelin-treated and eight saline-treated controls), blood samples were drawn in the morning before administration of buserelin or saline in week 1 and 4 of the injection treatment, and at sacrifice. During the 2 weeks after the fourth treatment session, GI transit time and galactose absorption were studied. Feces were collected during 12 h of fasting and analyzed for weight and fat content. After sacrifice, tissue samples from the stomach, ileum, transverse colon, and the distal part of the uterine horn were collected and rinsed in saline before fixation and processing for cryo- or paraffin-sectioning and histological evaluation. All methods are described in detail below.

### Blood and vaginal sampling

To study possible buserelin effects on the rat estrus cycle, vaginal smears and blood samples for hormone measurements were collected. On day 0 and 5 during session 1 and 4, vaginal smears were obtained using a cotton tipped applicator, rotated three times, 2 cm from the vaginal orifice. The vaginal smears were placed on microscope slides and stained with methylene blue and eosin for determination of the phase in the estrus cycle. The classification of the different cycles was performed according to established criteria [18,19]. Briefly, proestrus is characterized by a predominance of nucleated epithelial cells and estrus by anucleated, cornified epithelial cells. Metestrus is characterized by an equal portion of nucleated or anucleated epithelial cells and leukocytes, while diestrus is characterized by a predominance of leukocytes. Blood samples were collected from the tail vein, using Li heparin tubes (BD Microtainer, New Jersey, USA) and centrifuged at 3000 rcf (1.12 × R × (RPM/1000)^2^) for 5 min. Sera and plasma were harvested and stored at −20°C. Samples were collected at 9:00 am before the first injection of buserelin or saline (day 0), in the fourth session of injections (day 0 and 5), and at sacrifice. Vaginal smears and blood samples taken on day 0, before the first injection, served as an individual control for each rat.

### Tissue preparation

The gut segments and the uteri were opened and embedded, flattened, in filter paper. One portion of each gut segment was fixed in Stefanini’s fixative (a mixture of 2% formaldehyde and 0.2% picric acid in phosphate buffer, pH 7.2) for 22 h at 4°C, and the other portion and the uteri were fixed in 4% paraformaldehyde in 0.1 M phosphate buffer for 22 h at 4°C. Stefanini-fixed specimens were rinsed three times in Tyrode’s solution containing 10% sucrose, before being orientated and mounted for longitudinal- and cross-sectioning in Tissue-Tek (Sakura, Histolab, Gothenburg, Sweden), frozen on dry ice, and sectioned (10 μm). Paraformaldehyde-fixed specimens were dehydrated in ethanol, cleared in xylene, orientated for longitudinal- and cross-sectioning, embedded in paraffin, and sectioned (5 μm). Sections were processed for immunocytochemistry and histochemistry.

### Histochemistry

Measurements of wall layer thickness were performed on deparaffinized, hydrated, and hematoxylin-eosin-stained paraffin sections from the uterus by using a computerized, image-analyzing system (Imagescope, Aperio ScanScope GL SS5082, Vista, CA 92081, USA). The myometrial thicknesses to be measured were indicated manually, and then measured using a computerized binary cursor. Mean values of 6–10 representative measurements were calculated from each rat.

### Immunocytochemistry

For studies on enteric neuronal survival, antibodies against human neuronal protein HuC/D (HuC/D) were used as the general neuronal marker. Paraffin sections were deparaffinized, hydrated, and subjected to antigen retrieval by boiling in citrate acid buffer (0.01 M, pH 6) in a microwave oven (650 W) for 2 × 7 min. The sections were cooled and washed in distilled water followed by phosphate-buffered saline (PBS)/Triton. Sections were exposed to biotinylated, primary antibodies against HuC/D at 4°C overnight. For visualization of biotinylated HuC/D, a VECTASTAIN ABC kit containing horseradish peroxidase (HRP) and 3,3’-diaminobenzidine tetrahydrochloride (DAB) was used (Vector Laboratories, Inc., CA, USA). HuC/D-immunoreactive neurons stained dark brown and were counted in submucous and myenteric ganglia on longitudinally-cut sections using a computerized, image-analyzing system (Imagescope). The number of HuC/D neurons in colon was counted in scanned sections in a total length of at least 30 mm, cut at 6–9 different depths per region and rat. Synthetic antigens for testing the specificity of antibodies against HuC/D are not commercially available. Thus, omission of the primary antibodies was used as controls. Results are expressed as numbers of submucous or myenteric neurons, immunoreactive to HuC/D, per mm length of GI tract.

To study whether the neuronal loss was general or specific regarding the subpopulations of enteric neurons, the relative numbers of different subpopulations were studied in colon, as this was the most affected region in the former study [4]. Antibodies against CGRP, CART, galanin, GRP, NPY, NOS, 5-HT, somatostatin, SP, VIP, in combination with antibodies against HuC/D, were used on cryo sections. Since vesicular acetylcholine transporter (VAchT) immunoreactivity is mainly located on nerve fibers, antibodies against VAchT alone, and not in combination with HuC/D, were used on cryosections. Details on the antibodies are given in Table 1. Absorption controls were performed by adding an excess amount of antigen (10–100 μg of synthetic peptide diluted in antiserum) before exposure.

**Table 1:** **Details on antibodies**

**Raised against**	**Code no.**	**Host**	**Working dilution cryosections**	**Supplier**	**References**
CART (61–102)	H-003-61	Rabbit	1:5000	Phoenix, GmBH, USA	Ekblad et al. 2003; Zacharko-Siembida et al. 2014 [20,21]
CGRP	8427	Rabbit	1:5000	Euro-Diagnostica, Sweden	Ekblad et al. 1998 [8]
Galanin	8416	Rabbit	1:1000	Euro-Diagnostica, Sweden	Ekblad et al. 1988; Ekblad et al. 1998 [8,22]
GRP	R-6902	Rabbit	1:640		Ekblad et al. 1988 [22]
Hu proteins (HuC/HuD)	A-2127	Mouse	1:600	Life Technologies, USA	Lin et al. 2003 [23]
NOS	9223	Rabbit	1:5000	Euro-Diagnostica, Sweden	Ekblad et al. 1994; Kristensson et al. 2007 [24,25]
C-PON*	CA-08-300	Rabbit	1:3000	Genosys, UK	Ekblad et al. 1988; Kristensson et al. 2007 [22,25]
Serotonin (5-HT)	NSER	Rabbit	1:1200	Inc. Star Corp, USA	Mulder et al. 1997 [26]
Somatostatin	1758	Rabbit	1:3200	Kind gift from prof. J.J. Holst, Denmark	Ekblad et al. 1988; Kristensson et al. 2007 [22,25]
Substance P (SP)	SP7	Rabbit	1:800	Kind gift from prof. Emson, UK	Lindeström et al. 2002 [27]
VIP	7854	Rabbit	1:2000	Euro-Diagnostica, Sweden	Qian B et al. 2001; Kristensson et al. 2007 [25,28]
VAchT	AB1578	Get	1:2000	Chemicon, USA	Arvidsson U et al., 1997 [29]

*C-terminal flanking peptide of NPY (used for the detection of NPY-containing neurons).

The sites of the antibody-antigen reactions were visualized by exposure to a mixture of DyLight TM 488-conjugated goat anti-mouse IgG serum and Alexa Fluor TM 594-conjugated donkey anti-rabbit IgG serum, or solely to Texas red donkey anti-goat (all diluted 1:1000; Jackson ImmunoResearch Laboratories, Inc., Novakemi AB, Handen, Sweden), for 1 h in room temperature (RT) and then mounted in phosphate buffer:glycerol 1:1.

HuC/D-immunoreactive neurons also immunoreactive to NOS, 5-HT or any of the neuropeptides tested, were evaluated in cross- and longitudinally-cut, whole-wall sections. At least 150 submucous neurons and 250 myenteric neurons were counted for each set of double-stains and rat. The results are expressed as the percentage of HuC/D-immunoreactive neurons also immunoreactive to NOS, 5-HT or any of the neuropeptides. Since VAchT immunoreactivity is mainly located on nerve fibers, the relative number of VAchT-immunoreactive nerve cell bodies could not be evaluated. Nerve fiber density was evaluated on a 0, (+), +, ++, +++ scale, where 0 indicates no fibers, (+) = occasional fibers, + = few fibers, ++ = moderate numbers of fibers, and +++ = numerous fibers.

### Studies on gastrointestinal function

To measure total GI transit time, the rats fasted overnight by removing their food at 9:00 pm. At 9:00 am the next day, the rats were given a bolus dose (1 ml) of carbon suspension trough gavage administered orally to the stomach (150 mg/ml, Abigo Medical, Gothenburg, Sweden) before being placed in separate cages with free access to food and water. The rats were continuously and manually monitored by staff being present all the time, until the first carbon-containing fecal pellets were seen to be excreted.

In order to study fecal weight and fecal content [30], feces were collected and weighed from all rats during the 12 h of fasting, prior to the measurement of GI transit time, see above. The fecal samples were kept in open glass tubes and left to dry at RT for 3 months in a fume cupboard. The dry feces were ground in a mortar, transferred to a 20 ml glass vial, and weighed. To extract the fat from the feces, 10 ml of dichloromethane (DCM, Chromasolv, Sigma-Aldrich, Stockholm, Sweden) was added. The mixture was stirred vigorously for 1 h at RT. The DCM phase was filtered with a plastic syringe (10 ml), fitted with a polyethylene frit (20 μm, 10 ml, Biotage, Uppsala, Sweden) and a syringe filter (1 μm, Acrodisc glass fiber, Pall, NY, USA), and collected in a pre-weighed glass vessel. The extraction procedure was repeated twice for a total of 30 ml of the DCM phase. When executing the last extraction, the solid material and the DMC were transferred to the syringe. To ensure that as much as possible of the organic solvent was filtered into the glass vessel, a plunger was used to squeeze the solid material. The solvent was removed by evaporation and the residue (fat content) was weighed.

In order to study the absorptive capacity of galactose after 12 h of fasting, a bolus dose of 1 ml of 5% galactose dissolved in saline was given via a stomach tube as an oral bolus dose under anesthesia. Blood samples were collected in Li Heparin tubes from the tail vein using a neoflon catheter, before and 10 min, 30 min, and 90 min after the bolus dose [31]. Galactose levels in plasma were analyzed with a BioVision’s Galactose Assay Kit (K621-100, BioVision Incorporated, CA, USA) according to the manufacturer’s protocol. Briefly, galactose is oxidized using a galactose probe, an enzyme mix, and HRP, generating a colored product analyzed at the optic density of 570 nm.

### Serum analyses

Analyses of IgM- and IgG antibodies in serum against GnRH, GnRH receptor (GnRHR), LH, and LH receptor (LHR) were carried out in blood samples collected at sacrifice by an enzyme-linked immune sorbent assay (ELISA) as described in detail previously [3]. Briefly, the wells of microtiter plates (456537 Nunc, Roskilde, Denmark) were either coated with human GnRH or N-terminal GnRH-R peptide ((NH2)-ANSASPEQNQNHCSAINNSIPLMQGNLPY) conjugated with ovalbumin (OVA) (Innovagen, Lund, Sweden), 100 ng/well, in an overnight incubation at 4°C. Thereafter, the plastic wells were blocked with 0.5% bovine serum albumin (BSA) (A-7030, Sigma, St Louis, USA) in PBS (10 mM PO_4_^3−^, 137 mM NaCl, and 2.7 mM KCl, pH 7.4) containing 0.05% Tween-20 (PBS-T). The dilutions of patient serum (1:400 in 1.0 μg OVA (A-5503, Sigma)/ml 0.5% BSA in PBS-T) or mouse anti-human GnRH antibody (1.11 mg/ml, ab62432, Abcam, Cambridge, MA, USA) in serial dilution (1:2 000–1:32 000 to construct a standard curve) or rabbit anti-human GnRHR antibody (90217.09, Innovagen) in serial dilution (1:8 000–1:128 000) were then added to the plates and incubated for 2 h at RT. After rinsing with PBS-T, deposition of antibodies directed to GnRH or GnRHR was detected using the secondary antibodies rabbit anti-rat IgG-biotin (ABCAM102170, Abcam, Cambridge, MA, USA) or goat anti-rat IgM-biotin (ABCAM97178, Abcam) diluted 1:10 000 in PBS-T.

Analysis of antibodies against LH was carried out with intact, purified, native human LH (MBS537383, MyBiosource, San Diego, CA, USA), 100 ng/well, in PBS or only PBS-T (to provide an internal blank). After overnight incubation at 4°C, the plates were washed three times with PBS-T and blocked with 0.5% BSA in PBS-T. Dilutions of serum (1:200) from patients and blood donors, or rabbit anti-human LH antibody (MBS535386, MyBiosource) in serial dilution (to construct a standard curve), with BSA in PBS-T were then added to the plates in triplicate (two wells coated with LH and one well coated with PBS-T) and incubated for 2 h at RT. The washing procedure was repeated and deposition of autoantibodies directed to LH was detected using secondary antibodies as described above, appropriately diluted in PBS-T.

Analysis of antibodies against the LHR was carried out with the N-terminal LHR peptide ((NH2)-MKQRFSSALQLLKLLLLQPPLPRALC), conjugated with OVA (Innovagen), in 100 mM Carbonate buffer pH 9.2 or only Carbonate buffer (to provide an internal blank). After overnight incubation at 4°C, the plates were washed three times with PBS-T and blocked with 0.5% BSA in PBS-T. Dilutions of serum (1:200) from patients and blood donors with BSA in PBS-T were then added to the plates in triplicate, two to LHR and one to Carbonate buffer-coated wells, and incubated for 2 h at RT. The washing procedure was repeated and deposition of autoantibodies directed to LHR was detected using secondary antibodies as described above, appropriately diluted in PBS-T.

The absorbance at 405 nm was measured after 30 min of incubation at RT. Antibody levels are expressed as arbitrary units (AU) (absorbance values after subtracted background levels and multiplied with 1000).

### Plasma analyses

Due to the small amount of rat plasma, all analyses were performed with a single sample. Samples were analyzed according to the manufacturer’s instructions, and the resulting color changes were measured at the optical density of 450 nm. Each sample was interpolated from the standard curve.

Follicle-stimulating hormone (FSH)-, 17β estradiol (E2)-, and LH ELISA kits (MBS720215, MBS162143 and MBS161787, respectively, MyBiosource, San Diego, CA, USA) based on the double antibody sandwich technology were used in plasma samples collected at study start and during session 4. Plates were pre-coated with monoclonal antibodies selectively recognizing rat FSH, E2 or LH. Standards of FSH: 2.5, 5.0, 10, 25, and 50 ng/ml; E2: 30, 60, 120, 240, and 480 ng/l; and LH: 31.2, 62.5, 125, 250, 500, and 1000 ng/ml (to form a standard curve) or undiluted rat heparin plasma samples were then added to the wells on a microtiter plate (Nunc). A standardized preparation of immune complexes in the form of HRP-conjugated polyclonal antibody (FSH), or antibodies labeled with biotin/streptavidin HRP (E2 and LH), were added.

A rat zonulin ELISA kit (MBS753035, MyBiosource) based on the competitive enzyme immunoassay technique was used to study epithelial integrity in plasma harvested at sacrifice. The standards of 25, 50, 100, 250, and 500 ng/ml, undiluted rat heparin plasma, and buffer were incubated together with zonulin HRP conjugate on a plate pre-coated with monoclonal anti-zonulin antibodies.

### Statistical analyses

All results are presented as medians (interquartile ranges (IQR)), except weight, which is presented as mean ± standard error of the mean (SEM). Statistical analyses were performed by using the Mann–Whitney *U*-test. P < 0.05 was considered statistically significant.

## Results

### General characteristics

One rat in each group was terminated because of administration to the lung of the oral galactose solution, leaving 11 buserelin-treated (B) rats and seven saline-treated control (C) rats for the analyses. The body weight gain increased transiently in buserelin-treated rats in week 5 and 9 (p < 0.001; see Additional file 1). These time points coincided with the end of the buserelin sessions 2 and 3. The plasma levels of FSH were extremely low or undetectable in all rats (data not shown). The LH kit was unusable on rat plasma and serum, as no standard curve could be constructed. The estradiol levels in plasma were in the same range at start (Day 0, session 1; C = 52 (41–62) ng/L, B = 55 (46–58) ng/L), but during session 4, they were elevated on day 0 and 5 in the buserelin-treated group, reflecting that the hypothalamus-hypophys-gonadal axis was not suppressed by buserelin treatment in this setting (p < 0.05; for details see Additional file 2). At start (day 0, session 1), the vaginal smears indicated that all rats were unsynchronized in their estrus cycle (12 buserelin-treated and eight saline-treated controls). After the first week of treatment, the saline-treated rats were still unsynchronized, while the buserelin-treated rats were all in metestrus or diestrus. In the fourth week of injection treatment, the saline-treated rats were still unsynchronized on day 0 and 5, while the buserelin-treated rats were all in metestrus on day 0 and in diestrus on day 5 (see Additional file 3). The uterine muscle layer was thicker in the group of buserelin-treated rats compared to saline-treated controls (p < 0.05; see Additional file 4).

### Neuronal loss and neuronal subpopulations

**Figure 1:**
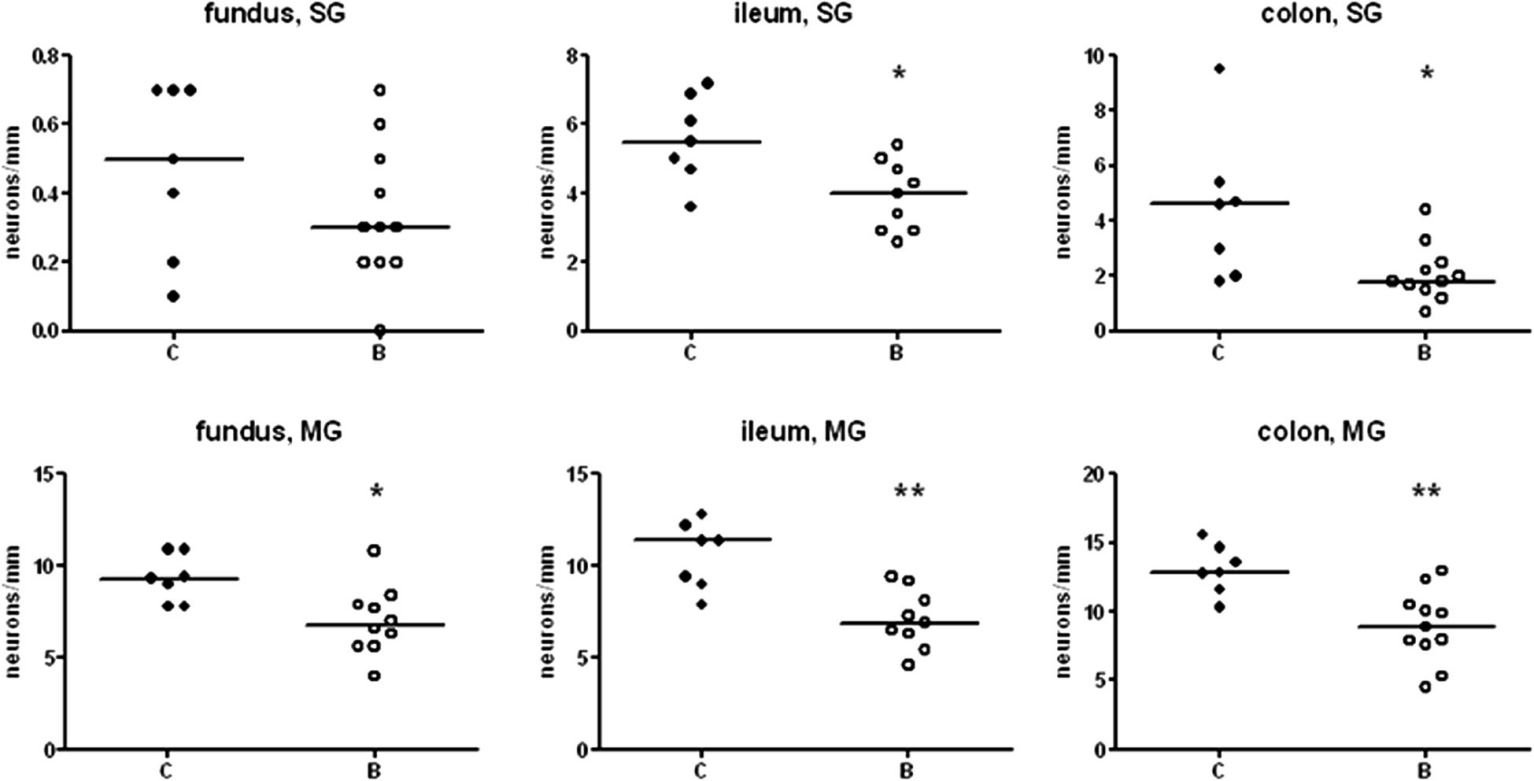
**Numbers of neurons in submucous (SG) and myenteric ganglia (MG) per mm length of section in stomach (fundus), ileum, and colon from rats treated with saline (C) or buserelin (B).** Rats treated with buserelin showed decreased numbers of submucous neurons in the ileum and colon, and myenteric neurons in the fundus, ileum, and colon. Cell-counting was performed on longitudinally-cut, whole-wall sections. Results are presented as individual data and medians. Statistical analyses were performed using the Mann-Whitney *U*-test, C = 7 rats and B = 9–11 rats. Statistical significance is indicated by *p < 0.05 and **p < 0.01.

Significant reductions in the absolute numbers of submucous neurons were detected in ileum and colon (27%, p < 0.05 and 61%, p < 0.05, respectively). Of the myenteric neurons, reduced numbers were noted in the stomach (27%, p < 0.05), ileum (39%, p < 0.01), and colon (31%, p < 0.01). The loss of neurons was statistically more pronounced in myenteric than in submucous ganglia, and more pronounced in the colon and ileum than in the stomach (Figure 1; for raw data see Additional file 5).

**Figure 2:**
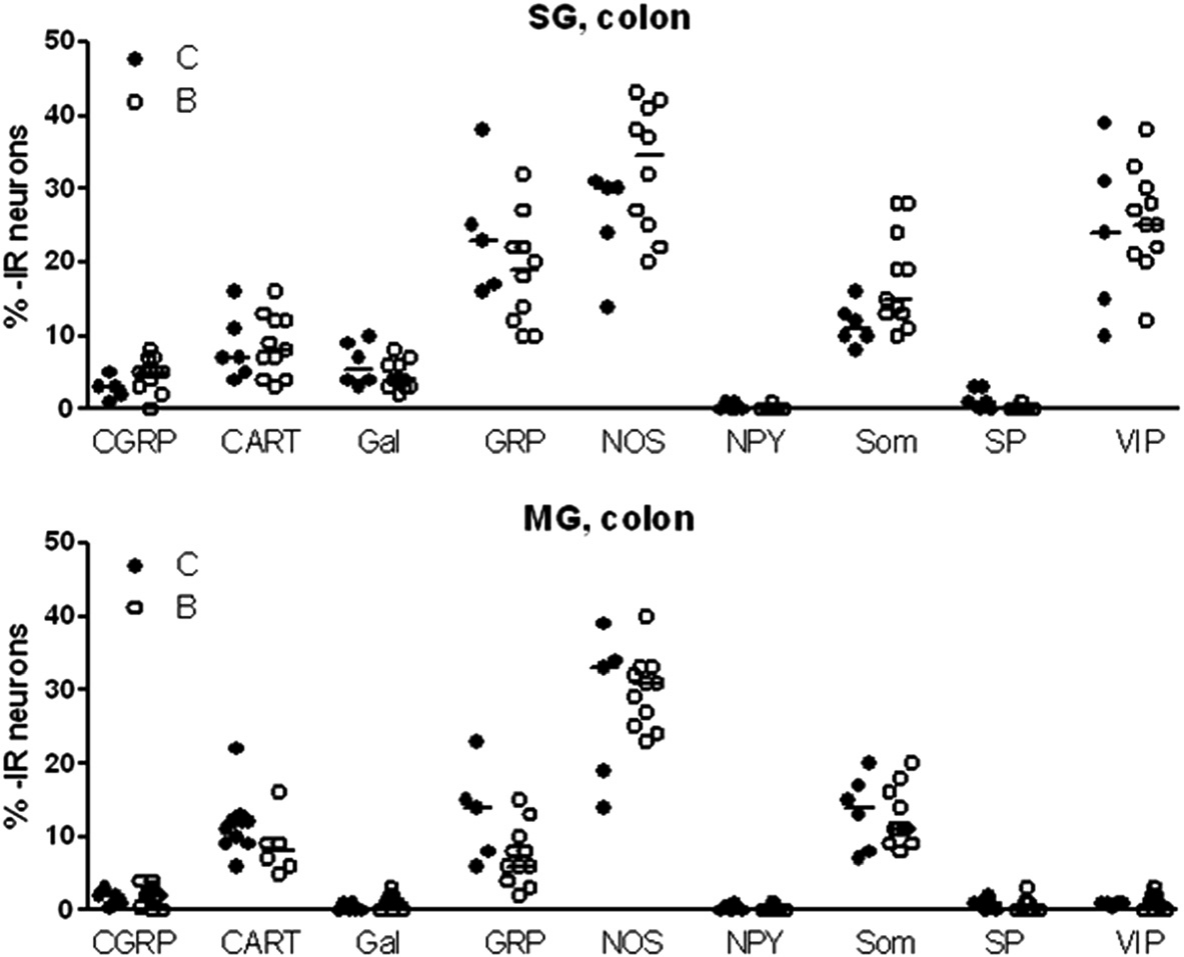
**Relative numbers of immunoreactive neurons in submucous (SG) and myenteric (MG) ganglia in colon for calcitonin gene-related peptide (CGRP), cocaine- and amphetamine-related transcript (CART), galanin (Gal), gastrin-releasing peptide (GRP), neuropeptide Y (NPY), nitric oxide synthase (NOS), somatostatin (som), substance P (SP), and vasoactive intestinal peptide (VIP), expressed as % of human neuronal protein HuC/D-immunoreactive neurons, from rats treated with four sessions of saline (C) or buserelin (B).** Cell-counting was performed on cross- and longitudinally-cut, whole-wall cryo sections, doubly immunostained for HuC/D and respective neuropeptide or NOS. With the exception of submucous som-immunoreactive neurons, which showed a tendency to increase after buserelin treatment (p = 0.062), no differences in any of the neuronal subpopulations were found between groups. Results are presented as individual data and medians. Statistical analyses were performed by the Mann-Whitney *U*-test, C = 5–6 rats and B = 10–11 rats.

**Figure 3:**
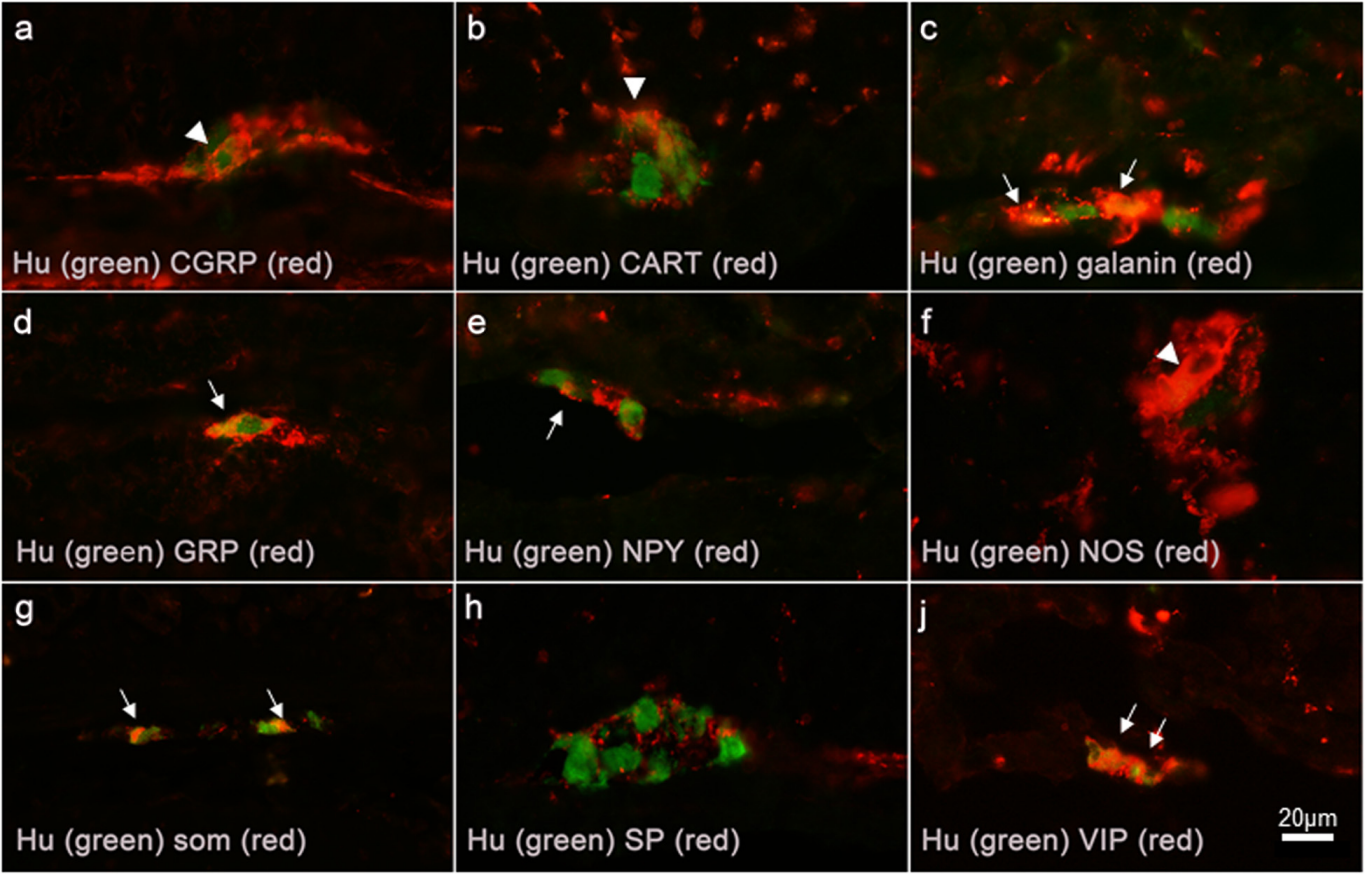
**Cryo sections of colon from rats treated with saline (b, c, e, g, j) or buserelin (a, d, f, h) immunostained with human neuronal protein HuC/D (green) and calcitonin gene-related peptide (CGRP), cocaine- and amphetamine-related transcript (CART), galanin, gastrin-releasing peptide (GRP), neuropeptide Y (NPY), nitric oxide synthase (NOS), somatostatin (som), substance P (SP), and vasoactive intestinal peptide (VIP)(red).** Micrographs are merged and show intense immunostaining, irrespective of treatment. Arrowheads indicate HuC/D-immunoreactive myenteric neurons also immunoreactive to CGRP **(a),** CART **(b),** and NOS **(f)**. Arrows indicate a HuC/D-immunoreactive submucous neuron also immunoreactive to galanin **(c),** GRP **(d),** NPY **(e)**, som **(g)**, and VIP **(j)**. In micrograph **(h)**, SP-immunoreactive fibers innervate a myenteric ganglia. Scale bar = 20 μm applies for all the micrographs in Figure 3.

In colon, a small number (fewer than 10%) of submucous nerve cell bodies were immunoreactive to CGRP, CART, galanin, NPY or SP, while GRP-, NOS-, and VIP immunoreactivity were found in 10%–43% of submucous neurons. The relative number of somatostatin-immunoreactive submucous nerve cell bodies was 11% in colon from controls, but showed a tendency to increase to 14% after buserelin treatment (p = 0.062; Figure 2; for raw data see Additional file 5). In both controls and buserelin-treated rats, few colonic myenteric nerve cell bodies were found to contain CGRP-, galanin-, NPY-, SP-, or VIP immunoreactivity, and moderate numbers of neurons were found to contain CART, GRP, and somatostatin, whereas myenteric NOS immunoreactivity was abundant (Figure 2). Nerve cell bodies immunoreactive to 5-HT was not detected, but a sparse network of nerve terminals was found in the mucosa and myenteric ganglia. The nerve fiber density was not affected by buserelin treatment in any of the neuronal subpopulations studied (Table 2). Pictures of immunostainings of NOS and neuropeptides studied are shown in Figure 3.

**Table 2:** **Nerve fiber density and distribution in colon was identical in saline- and buserelin-treated rats**

	**CGRP**	**CART**	**Gal**	**GRP**	**NPY**	**NOS**	**5-HT**	**Som**	**SP**	**VIP**	**VAchT**
**M**	(+)	++	++	++	++	+	(+)	+	(+)	+++	(+)
**SM**	(+)	+	++	++	+++	+	0	+	(+)	+++	(+)
**SG**	+	++	+++(+)	+++	++	++	0	+	(+)	+++	+
**MG**	+	+++	+++	+++	++	+++	+	+	++	++	++
**CM**	(+)	++++	+(+)	+	++	+++(+)	0	(+)	(+)	++	(+)
**LM**	(+)	+	(+)	+	++	+	0	(+)	(+)	+	+
**BV**	(+)	(+)	0	0	+++	(+)	0	+	0	(+)	0

The colon mucosa (M), submucosa (SM), submucous ganglia (SG), myenteric ganglia (MG) circular (CM) and longitudinal (LM) muscle layers, and blood vessels (BV) were evaluated separately. The density and distribution of nerve fibers immunoreactive to calcitonin gene-related peptide (CGRP), cocaine- and amphetamine-related transcript (CART), galanin (Gal), gastrin-releasing peptide (GRP), neuropeptide Y (NPY), nitric oxide synthase (NOS), serotonin (5-HT), somatostatin (Som), substance P (SP), vasoactive intestinal peptide (VIP), and vesicular acetylcholine transporter (VAchT) in colon of saline- and buserelin-treated rats are shown combined as no differences in nerve fiber density and distribution were found between groups. Nerve fiber density was evaluated on a 0, (+), +, ++, +++ scale, where 0 indicates no fibers, (+) = occasional fibers, + = few fibers, ++ = moderate numbers of fibers, and +++ = numerous fibers. n = 5–11.

### Studies on gastrointestinal function

**Figure 4:**
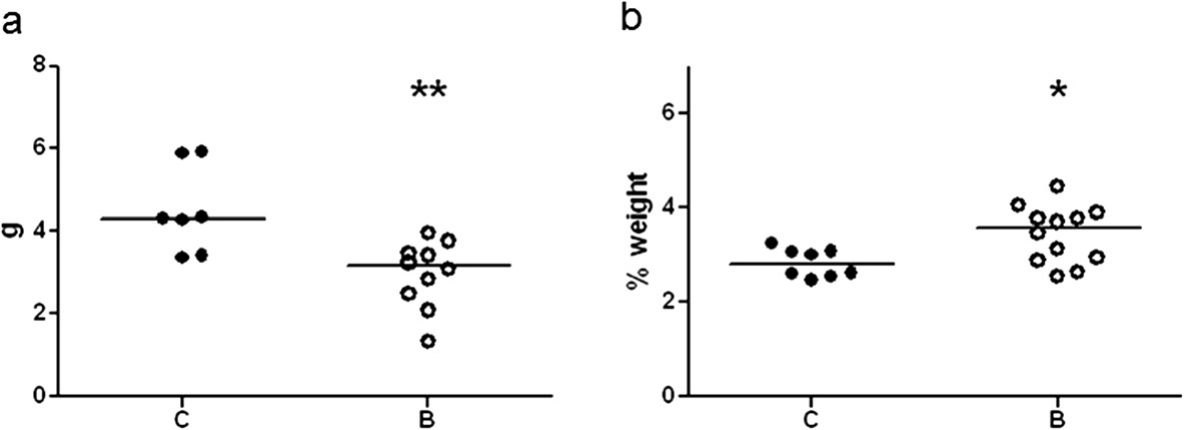
**Fecal weight (a) and fecal fat (b) in saline (C)- and buserelin (B)-treated rats.** Feces were collected and weighed after 12 h of fasting. Feces from buserelin-treated rats had a lower weight (g) compared to feces from saline-treated rats. Fecal fat was analyzed on dried feces. Buserelin-treated rats had an increased weight percentage of fat in feces compared to saline-treated rats. Results are presented as individual data and medians. Statistical analyses were performed by the Mann-Whitney *U*-test, C = 7–8 and B = 10–12 rats. Statistical significance is indicated by *p < 0.05 and **p < 0.01.

Total feces weight decreased in buserelin-treated animals, whereas total fat content per gram dried feces increased, compared to control rats (Figure 4a and b; for raw data see Additional file 5).

**Figure 5:**
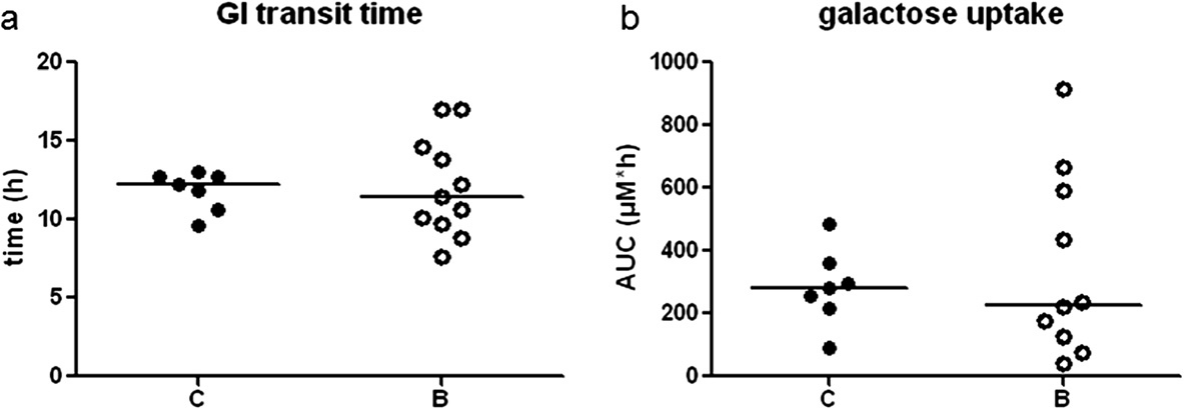
Gastrointestinal (GI) transit time (a) and total concentration of absorbed galactose (μM*h, area under curve (AUC)) (b) in saline (C)- and buserelin (B)-treated rats. No difference in total GI transit time was noted **(a)**. The AUC of galactose in plasma did not differ between the two groups **(b)**. Results are presented as individual data and medians. Statistical analyses were performed by using the Mann-Whitney *U*-test, C = 7 and B = 11 rats.

There was no difference in the total GI transit time between the buserelin-treated and the control rats, but a large spread of individual values was noted within the buserelin-treated group (Figure 5a). The area under the curve (AUC) of absorbed galactose did not differ between the two groups, but again, a large spread of individual values was noted within the buserelin-treated group (Figure 5b).

**Figure 6:**
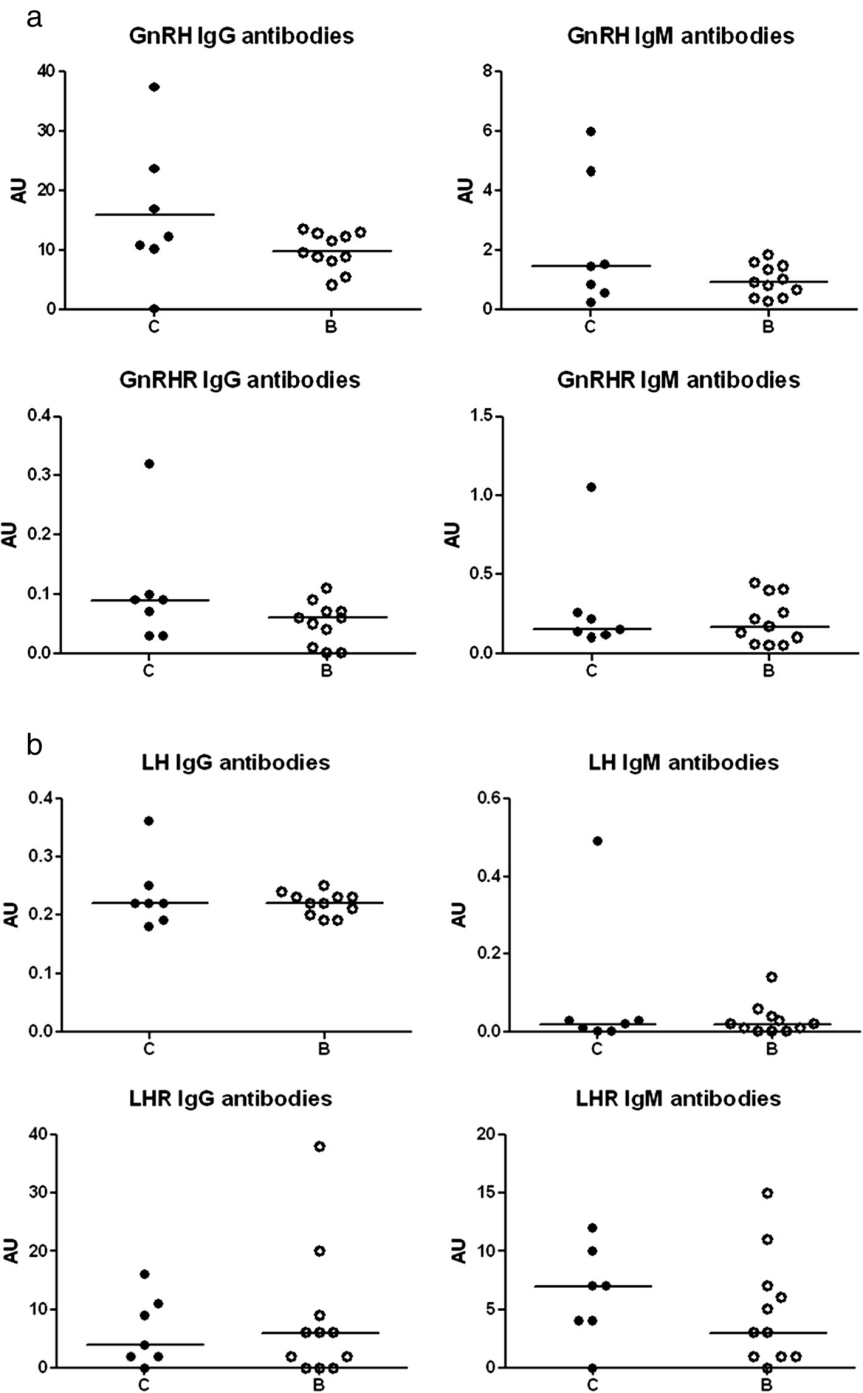
**Circulating antibodies. a**. Circulating IgG- and IgM antibodies against gonadotropin-releasing hormone (GnRH) and its receptor (GnRHR) in saline (C)- and buserelin (B)-treated rats. Low titers (arbitrary units (AU)) of antibodies were found in both saline- and buserelin-treated rats, with no differences between the groups. Results are presented as individual data and medians. Statistical analyses were performed by using the Mann-Whitney *U*-test, C = 7 and B = 11 rats. **b**. Circulating IgG- and IgM antibodies against luteinizing hormone (LH) and its receptor (LHR) in saline (C)- and buserelin (B)-treated rats. Low titers (arbitrary units (AU)) of antibodies were found in both saline- and buserelin-treated rats, with no differences between the groups. Results are presented as individual data and medians. Statistical analyses were performed by using the Mann-Whitney *U*-test, C = 7 and B = 11 rats.

Low titers of antibodies against GnRH, GnRHR, LH, and LHR were found in serum in both groups (Figure 6a and b). Plasma levels of zonulin were similar in both groups (C = 41 (32–49) ng/ml; B = 34 (33–38) ng/ml).

## Discussion

The present study confirms previous findings of a marked loss of enteric neurons (27%–61%) in rat appearing soon after buserelin administration [4], with a tendency of increased relative number of somatostatin-immunoreactive submucous neurons. Buserelin caused increased estradiol levels in plasma and thickened uterine muscle layers, indicating high estrogen activity. Furthermore, the study revealed that buserelin-treated rats had a decreased feces weight with higher fat content, compared to controls. In view of the marked buserelin-induced loss of enteric neurons, the differences between buserelin-treated rats and controls were surprisingly modest in the physiological parameters investigated. The neuronal loss described previously was most pronounced in colon [4], but in the present study, the neuronal loss was the same in ileum as in colon.

All rats had free access to food and water, except during two short fasting periods. The decreased feces weight after buserelin treatment is not easily explained by dehydration, changed food intake or cachexia, as the body weight was unaltered. Secretion of intestinal fluid is regulated mainly by submucous ganglia [32]. The tendency to relative increase in the number of submucous somatostatin neurons seen in the present study might affect intestinal secretion, since these most likely are cholinergic secretomotor neurons, also containing CGRP, issuing projections both orally and anally to the mucosa and submucosa [6,33]. Increased somatostatin inhibitory input could lead to reduced secretomotor activity, leading to reduced fluids in the lumen and feces, affecting fecal water content and fecal weight.

The most plausible explanation for the higher percentage of fat in the feces of buserelin-treated rats is maldigestion and/or malabsorption of dietary fat. The regulatory mechanisms of digestion and absorption of fat are complex, with several neural short and long reflexes involving an intricate synchronization of intestinal motility and secretion, gallbladder contraction, exocrine pancreas secretion, and hormone release from endocrine cells. An intact neural function is crucial for the regulation of gut peptide secretion from endocrine cells, and for emptying of bile and pancreatic enzymes into the intestinal lumen [34,35].

In the present study, no increase of the relative numbers of VIP- or NOS-immunoreactive neurons were found, when the rats were euthanized 2 weeks after the end of the fourth session. In our previous study of colon, a significant increase of myenteric VIP-containing neurons was found after two sessions of buserelin treatment, and in both submucous and myenteric NOS-immunoreactive neurons after four sessions of treatment, when rats were euthanized immediately after the fourth treatment [4]. This suggests that 2 weeks post treatment, the acute neuronal/tissue impingement has subsided, since increased expression of VIP and NOS are early signs of an injurious event, e.g. lipopolysaccharide (LPS) challenge, axotomy or after ischemia followed by reperfusion [7,10,11]. In general, the relative numbers of neurons immunoreactive to CGRP, CART, galanin, GRP, NPY, NOS, 5-HT, SP, VIP or VAchT, and their nerve fiber density, were unaffected by buserelin treatment. This implies that the neuronal loss is nonselective to subpopulations. It also strengthens the concept that ENS is highly adaptive and strives to maintain its original set of neuronal subpopulations, as well as its distribution and density of nerve terminals.

Although the enteric neuronal loss was most pronounced in the myenteric ganglia, the GI transit time and galactose absorption were generally not affected by repeated buserelin treatments. A greater inter-individual difference was seen in buserelin-treated rats, in analogy to the inter-individual effect on GI function observed in women after treatment with a GnRH analog [2,3,36]. The transport protein called sodium-glucose transport protein 1 (SGLT-1) is used by enterocytes to absorb both galactose and glucose, and the glucose transporter protein (GLUT2) is located basally for export [37,38]. The absorption of nutrients is a vital mechanism depending mainly on the function and integrity of the intestinal mucosa, while intestinal innervation plays a minor role, probably explaining the lack of effect on absorption in the current study.

Initial administration of a GnRH analog in man leads to increased secretion of FSH and LH, whereas continuous stimulation leads to receptor desensitization and down-regulation of gonadotropin secretion after 10 days of treatment [39]. The LH secretion in female rats starts in proestrus, peaks in the evening, resulting in ovulation, and shows low levels next morning (estrus). FSH- and LH secretions start simultaneously, but FSH remains elevated throughout the estrus [40,41]. The FSH- and LH levels are very low during the rest of the reproductive cycle [41], and are secreted in a pulsatile manner, like GnRH [42]. Although plasma FSH was not measureable in the present study, when the animals were in other cycles than proestrus and estrus, the combination of elevated estradiol levels in plasma, thickened uterine muscle layers, and synchronized reproductive phases in buserelin-treated rats indicate a GnRH influence on the hypothalamus, leading to enhanced FSH- and LH secretion with ensuing estradiol secretion. From another study, the buserelin-induced loss of enteric neurons is suggested to be mediated via LH receptor activation, since the relative number of LH receptor-expressing myenteric neurons was decreased after buserelin treatment, and neither GnRH nor its receptor could be identified in the ENS of the rat [4].

As there was no difference in the prevalence of circulating antibodies against GnRH, LH, and their receptors between groups, the noted enteric neuropathy is probably not mediated by autoantibodies. GnRH has been found in enteric neurons in man, and the expression of GnRH antibodies after buserelin-induced dysmotility is probably secondary to the marked neuropathy [1,2], as antibody prevalence and titer were the same before and after buserelin treatment [3]. Alpha- and beta estrogen receptors are expressed in enteric neurons [43], and theoretically, estradiol could exert degenerative effects on the ENS. However, estrogens are widely used in contraceptives and hormonal replacement therapies, without any reports of ensuing dysmotility. The mechanism behind the buserelin-induced neuropathy needs to be further evaluated, for example, in cell culture experiments using primary enteric neurons.

Zonulin participates in the physiological regulation of intercellular tight junctions in the small intestine. Dysregulation of the zonulin pathway may lead to intestinal disorders, and elevated zonulin levels in serum are reported in untreated celiac disease [44]. Similar plasma levels of zonulin in saline- and buserelin-treated rats, as found in the present study, suggest that gut mucosa integrity is mainly uncompromised by the buserelin-induced neuropathy.

In the daily clinical work, tests on nutrient absorption and analyses of fat in feces are never or seldom performed. The degree of fat malabsorption can be evaluated, but it is a rough estimation, and is primarily used to reveal exocrine pancreatic insufficiency or severe epithelial dysfunction, e.g. celiac disease. About half of the patients with GI complaints, consulting a gastroenterology ward, are diagnosed as suffering from IBS [45]. The etiology behind IBS is uncertain [46], and clinical investigations are unable to reveal objective parameters explaining the dysfunction. Affective disturbances and psychiatric disorders are common, concomitant diagnoses with IBS [45]. Examinations show that IBS patients are associated with an enhanced perception of personal vulnerability to illness [47], and they often over-report symptoms and suffer from somatization disorders [48,49]. Functional magnetic resonance imaging (fMRI) reveals significant differences in the neural processing of pain between IBS patients and controls, further underlining the importance of psychological factors in the pathophysiology of visceral hypersensitivity in these patients [50,51]. The results in the present study make the possibility feasible that severe, unrevealed enteric neurodegeneration exists in subgroups of IBS patients. Different etiologies for the GI symptoms may explain the difficulties to find conclusive explanations in scientific reports. Chronic abdominal complaints may be a stress factor for the patient, leading to an abnormal central processing of the pain [50,51], and thus, psychological and cognitive dysfunctions in IBS may be secondary, rather than causal. A tendency of an increase in the relative number of somatostatin-immunoreactive submucous neurons was found in our present study. This is interesting since the transient receptor potential vanilloid 1 (TRPV1), which is involved in visceral pain signaling, is up-regulated in IBS patients where it stimulates increased release of somatostatin and SP [52]. Patients who had undergone several IVFs with buserelin treatments suffered from more abdominal pain and exacerbation of IBS 5 years after than prior to treatment, although no obvious dysmotility was found [3]. As the ENS seems to have a huge reserve of functional capacity, the treated patients may acquire a modest, but subclinical, enteric neuropathy making them more vulnerable to complications in, for instance, diabetes mellitus and neurological diseases.

## Conclusion

A marked enteric neuronal loss with modest effects on GI function is found after buserelin treatment. Increased feces fat content is suggested an early sign of dysfunction.

## Additional files

Additional file 1:
**Body weights.** Body weight over time studied on rats treated with one to four sessions with saline (C, −○) or buserelin (B, −●). One session consists of 5 days of treatment, with one daily subcutaneous injection of saline or buserelin, followed by 3 weeks recovery. All rats were healthy and gained weight throughout the experimentation. At the end of the second and third treatments (weeks 5 and 9), buserelin-treated rats showed a transient increase in weight compared to saline-treated rats. Results are presented as means and standard error of the mean (SEM) and analyzed by Mann-Whitney *U-* test, C = 7 and B = 12. Statistical significance is indicated by ***p < 0.001. Additional file 2:
**Plasma estradiol levels.** Estradiol (E2) plasma levels in rats, day 0 and 5, of the fourth treatment session with saline (C) or buserelin (B). The rats receiving buserelin had already high levels of estradiol, at start of the last treatment session (session 4) compared to saline-treated rats (p < 0.05). Estradiol levels were still high day 5 in buserelin- compared to saline-treated rats (p < 0.05), indicating a sustained buserelin-induced high estrogen activity. Results are presented as medians and analyzed by Mann-Whitney *U* -test, C = 6 and B = 12. Statistical significance is indicated by *p < 0.05. Additional file 3:
**Vaginal smear characterization.** Effects of buserelin or saline in individual rats on their estrus cycle. Vaginal smears were collected day 0 and 5 during session 1 and 4. The classification of the different phases in estrus cycle were performed according to established criteria (Freeman 2006, Maldonao-Devinci 2010): - Proestrus (Pro) is characterized by the predominance of nucleated epithelial cells. - Estrus (E) is characterized by the predominance of anucleated cornified cells. - Metestrus (M) is characterized by an equal portion of nucleated and anucleated epithelial cells and leukocytes. - Diestrus (D) is characterized by the predominance of leukocytes. Additional file 4:
**Thickness of uterine muscle layers.** Thicknesses of uterine muscle layer in rats treated with four sessions of saline (C) or buserelin (B). Buserelin-treated rats had hypertrophic uterine muscle layers compared to saline-treated rats. Results are presented as medians and analyzed by Mann-Whitney *U-* test, C = 5 and B = 12. Statistical significance is indicated by p < 0.05, which was considered statistically significant. Additional file 5:
**Raw data.** The number of enteric neruons in submucous ganglia (SG) and myenteric ganglia (MG) of fundus, ileum, and colon. The values of feces weight (g) and fat content (% of dried feces) are given. The number of immunoreactive neurons in submucous ganglia (SG) in colon for somatostatin (som), expressed as % of human neuronal protein HuC/D-immunoreactive neurons, from rats treated with four sessions of saline (controls) or buserelin. Link to the journal guidelines: http://www.biomedcentral.com.

## Abbreviations

CGRP: Calcitonin gene-related peptide
CART: Cocaine- and amphetamine-related transcript (CART)
ED: Enteric dysmotility
ENS: Enteric nervous system
E2: 17β estradiol
FSH: Follicle-stimulating hormone
Gal: Galanin
GRP: Gastrin-releasing peptide
GnRH: Gonadotropin-releasing hormone
HuC/D: Human neuronal protein HuC/D
IBS: Irritable bowel syndrome
LH: Luteinizing hormone
MG: Myenteric ganglia
NOS: Nitric oxide synthases
NPY: Neuropeptide Y
Som: Somatostatin
SG: Submucous ganglia
SP: Substance P
VIP: Vasoactive intestinal peptide
VAchT: Vesicular acetylcholine transporter
5-HT: Serotonin
