# Supplementary figures and images for: Structural and functional consequences of buserelin-induced enteric neuropathy in rat
Source: BMC Gastroenterol. 2014 Dec 11;14:209. doi: 10.1186/s12876-014-0209-7 (PMC4275936; doi:10.1186/s12876-014-0209-7)

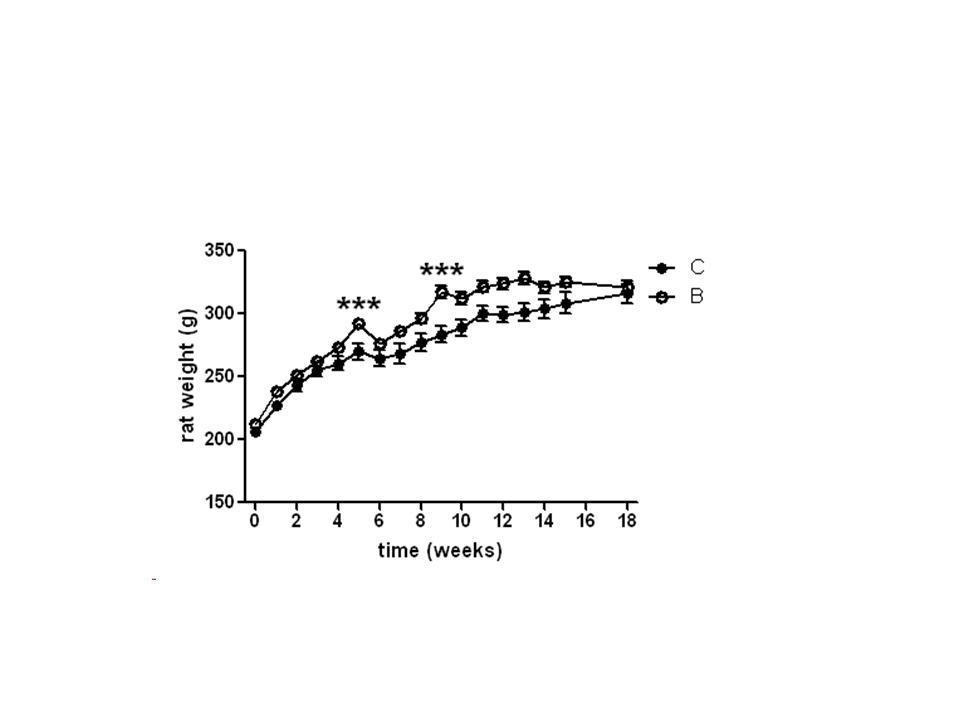

Supplement: Additional file 1: — Body weights. Body weight over time studied on rats treated with one to four sessions with saline (C, −○) or buserelin (B, −●). One session consists of 5 days of treatment, with one daily subcutaneous injection of saline or buserelin, followed by 3 weeks recovery. All rats were healthy and gained weight throughout the experimentation. At the end of the second and third treatments (weeks 5 and 9), buserelin-treated rats showed a transient increase in weight compared to saline-treated rats. Results are presented as means and standard error of the mean (SEM) and analyzed by Mann-Whitney U- test, C = 7 and B = 12. Statistical significance is indicated by ***p < 0.001. [file 12876_2014_209_MOESM1_ESM.tiff]

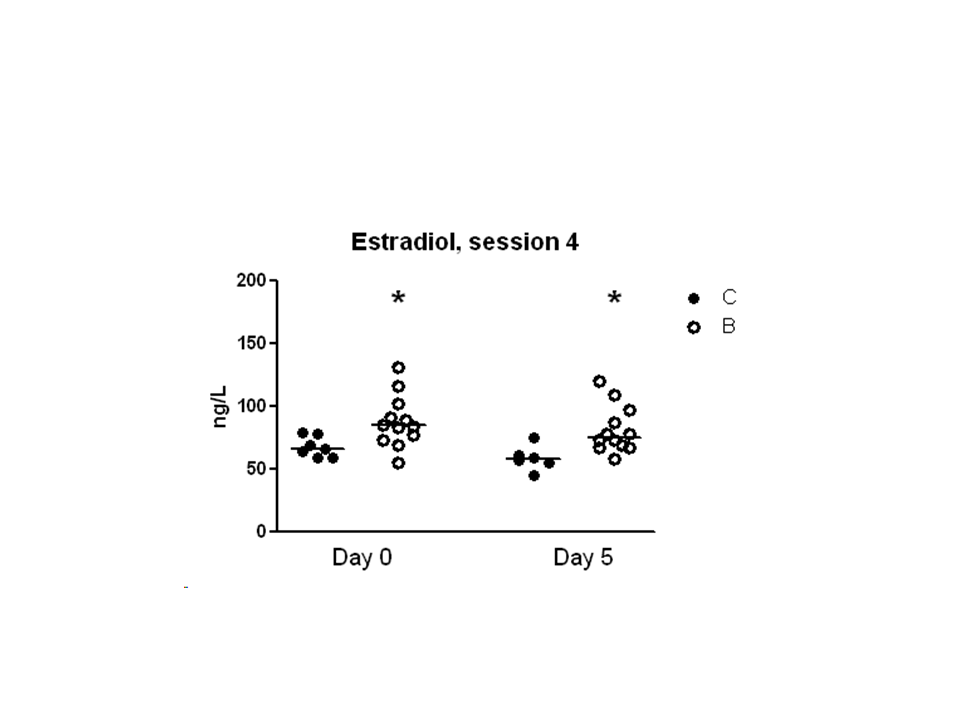

Supplement: Additional file 2: — Plasma estradiol levels. Estradiol (E2) plasma levels in rats, day 0 and 5, of the fourth treatment session with saline (C) or buserelin (B). The rats receiving buserelin had already high levels of estradiol, at start of the last treatment session (session 4) compared to saline-treated rats (p < 0.05). Estradiol levels were still high day 5 in buserelin- compared to saline-treated rats (p < 0.05), indicating a sustained buserelin-induced high estrogen activity. Results are presented as medians and analyzed by Mann-Whitney U -test, C = 6 and B = 12. Statistical significance is indicated by *p < 0.05. [file 12876_2014_209_MOESM2_ESM.tiff]

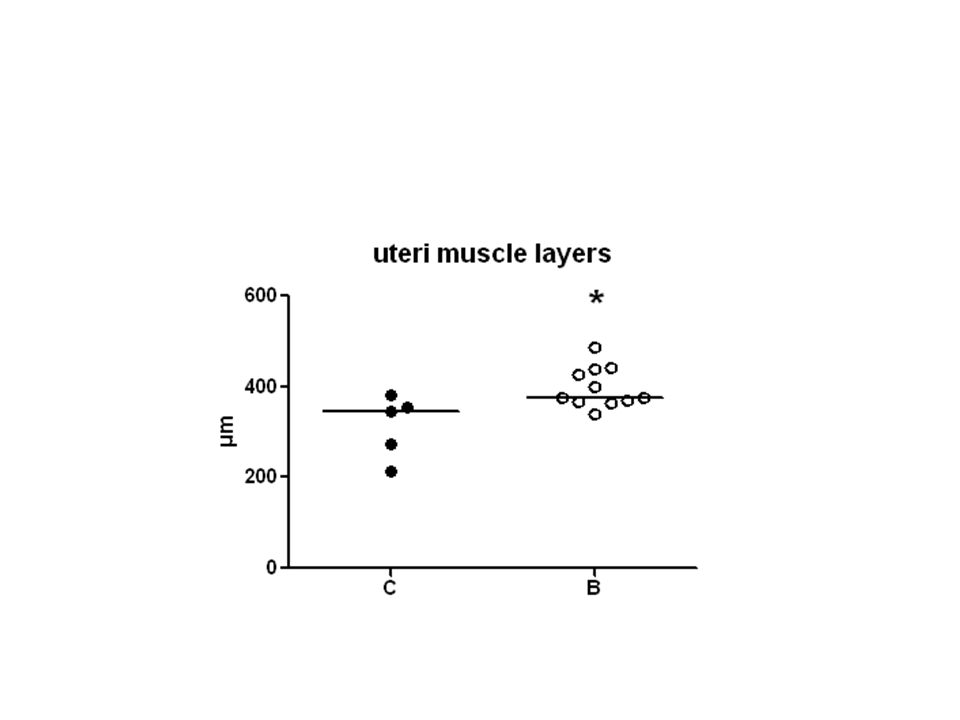

Supplement: Additional file 4: — Thickness of uterine muscle layers. Thicknesses of uterine muscle layer in rats treated with four sessions of saline (C) or buserelin (B). Buserelin-treated rats had hypertrophic uterine muscle layers compared to saline-treated rats. Results are presented as medians and analyzed by Mann-Whitney U- test, C = 5 and B = 12. Statistical significance is indicated by p < 0.05, which was considered statistically significant. [file 12876_2014_209_MOESM4_ESM.tiff]
