# Supplementary material for: Structural and functional consequences of buserelin-induced enteric neuropathy in rat
Source: BMC Gastroenterol. 2014 Dec 11;14:209. doi: 10.1186/s12876-014-0209-7 (PMC4275936; doi:10.1186/s12876-014-0209-7)
Supplement: Additional file 3: — Vaginal smear characterization. Effects of buserelin or saline in individual rats on their estrus cycle. Vaginal smears were collected day 0 and 5 during session 1 and 4. The classification of the different phases in estrus cycle were performed according to established criteria (Freeman 2006, Maldonao-Devinci 2010): - Proestrus (Pro) is characterized by the predominance of nucleated epithelial cells. - Estrus (E) is characterized by the predominance of anucleated cornified cells. - Metestrus (M) is characterized by an equal portion of nucleated and anucleated epithelial cells and leukocytes. - Diestrus (D) is characterized by the predominance of leukocytes. [file 12876_2014_209_MOESM3_ESM.pdf]

Session 1				Session 4			
**Controls (C)**		**Buserelin (B)**		**Controls (C)**		**Buserelin (B)**	
Day 0	Day 5	Day 0	Day 5	Day 0	Day 5	Day 0	Day 5
E	D	M	M	M	Pro	M	D
E	E	E	M	E	D	M	D
Pro	Pro	E	M	E	D	M	D
-	-	E	D	D	D	M	D
E	Pro	E	M	Pro	Pro	M	D
M	E	Pro	M	E	M	D	D
M	M	Pro	M	Pro	M	M	D
Pro	-	Pro	M	E	M	M	D
		E	M			M	D
		E	M			M	D
		M	D			M	D
